# Effects of Low-Immersive vs. High-Immersive Exercise Environment on Postural Stability and Reaction and Motor Time of Healthy Young Adults

**DOI:** 10.3390/jcm12010389

**Published:** 2023-01-03

**Authors:** Julia Ciążyńska, Janusz Maciaszek

**Affiliations:** Department of Physical Activity and Health Promotion Science, Poznan University of Physical Education, 61-871 Poznan, Poland

**Keywords:** health, virtual reality, head-mounted display, exergaming, high-immersive, low-immersive, physical exercise, health-related fitness, modern exercise, immersion

## Abstract

(1) Background: Many young adults spend their time playing games and watching television. This type of spending time should be used effectively, so it’s worth adding exercise and immersion to them. Bearing in mind that the video games and physical exercise also improve postural stability, motor time (MT) and reaction time (RT), it is worth reaching for new technologies with immersion that are widely available and can be used, for example, as a remote intervention. This study aimed to compare the effects of a low vs. high-immersive exercise environment on postural stability, RT and MT in young adults. (2) Methods: Ninety-three participants were randomly divided into a control group (CG; *n* = 48) and experimental group (EG; *n* = 45). The CG exercised according to the Tabata self-made video display on a television set, and the EG exercised according to the Audio Trip exergame. In addition to the postural stability, RT and MT, we monitored the heart rate, breath rate and energy expenditure for safety reasons and to note any differences. (3) Results: Significant differences were observed for both groups in RT (F(2.182) = 3.14, *p* = 0.046, η^2^ = 0.03) and MT (F(2.182) = 3.07, *p* = 0.049, η^2^ = 0.03) and in postural stability in eyes closed (EC): F(2.182) = 3.66, *p* = 0.028, η^2^ = 0.04 and eyes open in one leg (EO-OL): F(2.182) = 5.814, *p* = 0.04, η^2^ = 0.07. (4) Conclusions: The inclusion of a higher immersion produces greater improvements in RT, MT. Additionally, after a low-immersive exercise environment, participants have higher center of pressure (COP) path length values with EC and EO-OL tests, which testifies to less postural stability. Regarding COP trajectory, a smaller area surface means better performance for high-immersive participants after 30 min of exercise.

## 1. Introduction

Postural stability is a determinant of functional independence and has a significant role in the everyday life. As health behaviors, regular physical activity and exercise activate different muscle groups enhance postural stability [1,2,3,4,5], many young adults prefer spending time watching television and playing video games, which is an indicator of a sedentary lifestyle [6]. Researchers need to be well-acquainted and also promote modern possibilities to encourage exercise in young people. Bearing in mind that video games and physical exercise also improve postural stability, motor time (MT) and reaction time (RT) [7,8], it is worth reaching for new technologies with immersion that are widely available and can be used, for example, as a remote intervention [9,10,11,12]. There is a division in low, medium, and high immersion technologies. The researchers mention VR as a high-immersion type of experience and watching television or looking at a monitor screen as a low-immersion type of experience [13]. Immersion may be strongly connected to media form, that is, the properties of the technological system used to mediate the experience [14]. Immersion is the fundamental concept of the facilitation of emotion in a virtual environment [15,16], and felt emotions and a sense of reward can influence the performance of tasks [17]. Therefore, it can be assumed that higher immersion means higher satisfaction and the possibility of achieving better results in everyday tasks like postural stability test, RT or MT.

This study aimed to compare the effects of a low- vs. high-immersive exercise environment on postural stability, RT and MT in young adults. It was hypothesized that: 1. High-immersive exercise environment would produce greater improvements in postural stability than low-immersive exercise environment. 2. A high-immersive exercise environment would produce greater improvements in reaction and motor time than low-immersive exercise environment.

## 2. Materials and Methods

### 2.1. Participants and Study Design

**Figure 1:**
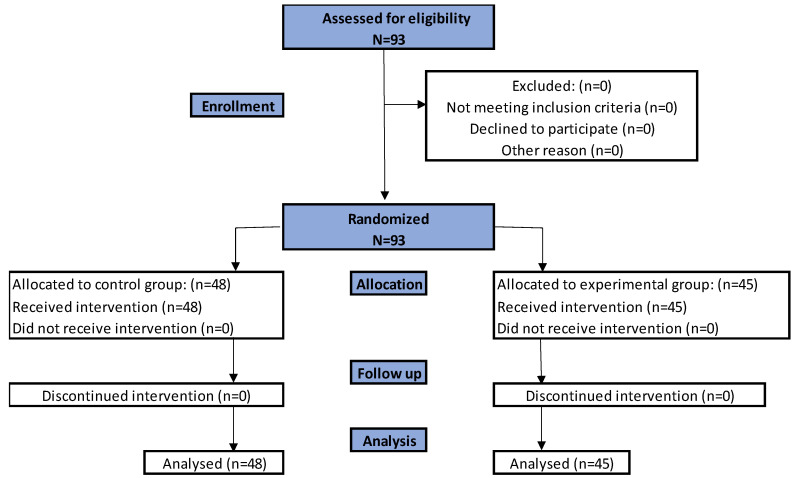
Flowchart for enrolment, randomization, and follow-up of study participants.

The study was designed as a randomized controlled trial with an intervention period of 1 day (2 × 30 min session of exercise). An a priori power analysis was conducted utilizing G*Power (Version 3.1.2; Heinrich Heine Universität, Dusseldorf, Germany). Sample size estimations were done for all analyses and the final targeted sample size was selected such that the analysis requiring the largest number of participants can be adequately powered. Based on meta-analyses, we expect a medium effect size (f^2^ = 0.14). The sample size required to reach a level of significance of 0.05 with a power of 0.80 is 83 participants. By making three measurements, considering two groups and an assumed dropout rate of 10%, we needed to recruit 92 people to test our hypotheses. A total of 93 participants were randomly assigned to the CG or EG. Flowcharts for enrolment, randomization, and follow-up of study participants are available in Figure 1. All participants completed the study.

To be eligible for participation in the study, the individual had to be a physical education student, be between 19 to 29 years old, have good health conditions (no neurological disorders, lack of disability, lack of mental disorders, no psychotropic medicines and lack of injuries and fresh injuries), have 10 on a scale or less in Ruffier Squat Test, and haven’t had interactions with VR equipment or similar VR stimulation before. Individuals were invited to participate in the study through personal invitations and emails. All participants provided signed informed consent, and the respective ethical committees granted ethical approvals for this study.

**Figure 2:**
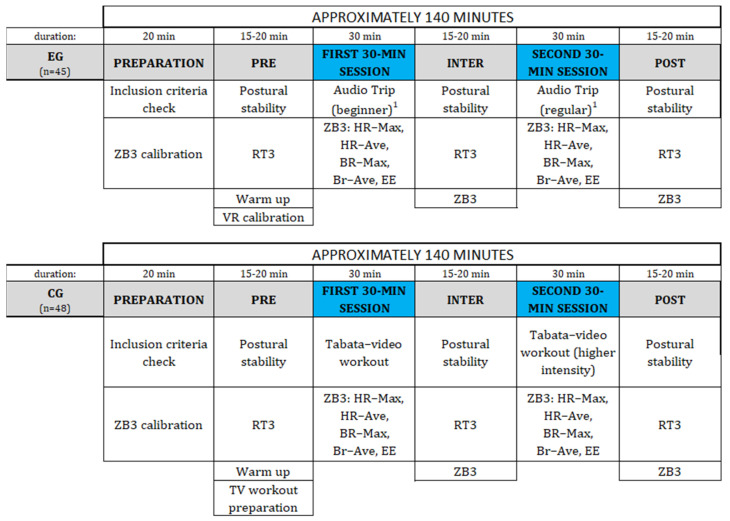
Study protocol presented in chronological order from left to right for EG and CG (Abbreviations: CG: control group; EG: experimental group; EE: energy expenditures; RT3: RT Test form S3; ZB3: Zephyr Bioharness 3 sensor). ^1^ level of difficulty.

After an inclusion criteria examination, the participants were randomized into an EG or a CG. The principal investigator carried out the randomization using a computer-generated random number table. The study period for EG and CG was approximately 140 min including: two 30-min session and pre, inter and post measurements. The study protocol is presented in chronological order in Figure 2.

The measurement test was supervised by one constant person; that is, it did not change either in the EG or in the CG. The conditions of the measurement did not differ from each other for both groups and respondents; the tests took place in the same room adapted to physical exercises, with room temperature from 19–22 °C in the autumn–winter period. On one day, 4 people were tested in the midday hours, two as a CG and two as an EG. One person who was unable to see the previous participants entered the study at the same time.

The reliability of the tests were increased by applying two postural stability measurements for each position in random order for the measurement with eyes open in free position (EO), measurement with eyes closed in free position (EC) and measurement with eyes open in one-leg position (EO-OL); applying pre-measurement for each group for RT, MT, EO, EC and EO-OL and rigorous standardization.

No significant differences (*p* > 0.05) between the group have been confirmed. The mean age of EG participants (*n* = 45) was 21.69 years (range: 19–28 years, SD = 2.76). The mean body weight was 71.03 kg (range: 44.5–106.5, SD = 13.07,). The mean body height was 173.98 cm (range: 155.6–186.1, SD = 8.09,). The mean Body Mass Index (BMI) was 23.3 (range: 18.4–32.3, SD = 3.23). According to the table for BMI [18], the EG had the following results:

- >18.5—1 participant
- 18.5–24.9—31 participants
- 25.0–29.9—13 participants

The EG was mixed; females constituted 48.9% (*n* = 22).

The mean age of CG participants (*n* = 48) was 22.25 years (range: 19–28 years, SD = 1.66). The mean body weight was 68.39 kg (range: 45.0–99.0, SD = 12.96). The mean body height was 173.83 cm (range: 159.4–191.2, SD = 8.63). The mean BMI was 22.5 (range: 17.3–29.5, SD = 2.80). According to the table for BMI [18], the CG had the following results:

- >18.5—1 participant
- 18.5–24.9—37 participants
- 25.0–29.9—10 participants

The CG was mixed; females constituted 54.2% (*n* = 26).

The participants’ characteristics are described in Table 1.

During the Ruffier squat test, subjects are made to do 30 squats in 45 seconds. The HR is recorded before the test (P1), at the end of 45 seconds (P2), and 1 minute after the test (P3). The test score is calculated in the form of an index—the Ruffier’s Index (RI) expressed as (P1 + P2 + P3) − 200)/10. The range of the RI given was 0 to 17, but higher than 10 is an adaptation to insufficient effort or even poor adaptation. The individual ranges are as follows: 0 is an exceptionally good adaptation to effort, between 0 and 5 is a good adaptation to effort, and between 5 and 10 (including 10) is the ability to adapt to the average effort. Ruffier originally developed this test for testing European subjects.

### 2.2. Hardware and Software

The VR Oculus Quest 64 GB system (Oculus Quest system software, Facebook Inc., released on 21 May 2019, Cambridge, MA, USA) was used as the high-immersive VR technology in this study, consisting of a wireless headset through which the VR environment could be viewed and played, two hand controllers that enabled interaction with the VR environment. People wearing corrective glasses could also take part in the study because a special overlay for glasses was applied. To ensure proper performance, the room size should be at least 2.0 m × 2.0 m. The VR Oculus Quest 64 GB has display 5.7 inches, resolution 2880 × 1600 px, reference resolution 1440 × 1600 px, OLED display type, refresh frequency 72 Hz. The name of the game which was used is Audio Trip and was distributed from Oculus Quest store app.

The POCO X3 NFC phone (MIUI 12.0.8) was used to create a video for the warm-up and tabata-workout. Music for warm-up video and tabata-workout are converted from YouTube to mp3 version. In both cases, a 42-inch flat-screen television set was used to run the videos.

### 2.3. Measure

Five methods were used for the usability evaluation: tabata-workout video on television display (low-immersive environment), Audio Trip VR game (high-immersive environment), Zephyr measurement, AMPI force platform and Vienna Test System.

**Figure 3:**
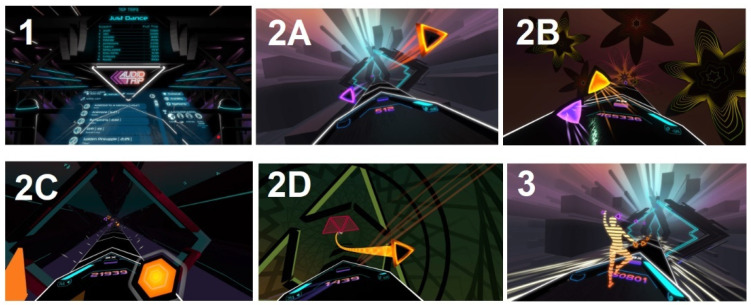
View from the Audio Trip game, from left: (**1**). Selecting a track from the list. (**2**). To the rhythm of the music, hitting two-colored triangles as intended: (2**A**). touching by R/L hand (2**B**). hitting at a certain angle by R/L hand (2**C**). smashing the drums by R/L hand (2**D**). following the path by R/L hand and dodging red barriers with whole body movement. (**3**). Player avatar view (back perspective).

The purpose of the Audio Trip game (Andromeda Entertainment 2019, Early Access 24 October 2019, Austin, TX, USA) is to dance to the rhythm and immerse into the virtual music and fitness environment. A player was obliged to ride ribbons, catch two colored gems, smash drums, and dodge virtual barriers. In this study, players from EG had to follow a special 30 min playlist two times on the beginner and regular mode. A playlist is a proprietary idea in which the beats per minute increase during play and the difficulty level in the second session increases, and thus the intensity increases. Details of the playlist are shown in Table 2. The view from the perspective of the gamer is shown in Figure 3.

The tabata-workout video, for CG, was a set of exercises created for the purposes of the described research. To make this video more similar to the VR exergame mentioned above, the same duration, the same playlist, and very similar types of movements were adjusted. Participants had to follow a special 30 min playlist for two times, imitating the trainer from the video. Details of the playlist are shown in Table 3.

A portable wireless piezoelectric recording system (Bioharness 3, Omnisense 3.9.7, Zephyr Technology Corp., Annapolis, MD, USA) was used to monitoring: energy expenditure—estimated according to the formula: EE (kcal) = Gender × (−55.0969 + 0.6309 HR + 0.1988 Weight + 0.2017 Age) + (1 − Gender) × (−20.4022 + 0.4472 HR − 0.1263 Weight + 0.074 Age). Gender—1 for male, 0 for female; heart rate (HR): including HR A (average) and HR M (maximum); breathing rate (BR): including BR A (average) and BR M (maximum).

To estimate postural stability, COP data were collected using the force platform (AMPI PJB-101 model, AMTI, Waterdown, MA, USA). The duration of a single test during static stance was 30 s, which is an appropriate time to record a reliable COP measure [19]. Therefore, the most commonly used parameter for analysis was the COP path length, which determines the distance traveled by COP (mm). The path length is known to be a reliable and valid measure of standing balance [20]. A shorter path length means better postural stability. The second metric is sway area, and it is defined as the Area95 or area of the 95% confidence ellipse around the COP trajectory (mm^2^). This metric allows assessing the size of the area of the COP movement on a force plate. A smaller area surface means a better performance [21]. The following measurements were conducted: EO, EC, EO-OL. In addition, the Romberg index was calculated from these tests. Values that are higher than 1 indicate greater oscillation with the eyes closed. Zero or negative values mean lower postural oscillation with the EC [22].

To study psychomotor, the mean MT and mean RT were both measured with the Reaction Time test (RT test form S3—Choice reaction yellow/tone, Vienna Test System, Schuhfried, Austria). This test required MT and RT movements, suitable for one finger of the right or left hand. When the rest button is used, the RT is the time between the start of the relevant stimulus and the moment the finger leaves the rest button. The score for mean RT is the RT per second. A high score indicates that in comparison to the reference population the respondent has an above-average ability to react quickly in response to relevant stimuli or stimulus constellations. The MT is the time that elapses between the moment the finger leaves the rest button and the moment contact is made with the reaction button when a relevant stimulus has been presented. This score provides information about the respondent’s speed of movement. A high score indicates that in comparison to the reference population the respondent has an above-average ability to implement a planned course of action quickly in reaction situations.

### 2.4. Statistical Analysis

Statistical analyses were performed using Statistica 13.3 (TIBCO Software Inc., Palo Alto, CA, USA) and statistical significance was defined as *p* ≤ 0.05. A Levane test was used to check the homogeneity of variance. A series of ANOVAs (groups: EG vs. CG) with repeated measures (pre, inter and post measurements) were performed to determine if significant differences occurred in the study outcomes. The effect size was measured by partial eta squared (η^2^). In the study, small, medium and large effect sizes were designated as η^2^ = 0.01, η^2^ = 0.06 and η^2^ = 0.14, respectively.

## 3. Results

### 3.1. Reaction Time and Motor Time

The RT Test form S3, choice reaction yellow/tone is a test for the assessment of RT for audible and visual stimuli.

**Figure 4:**
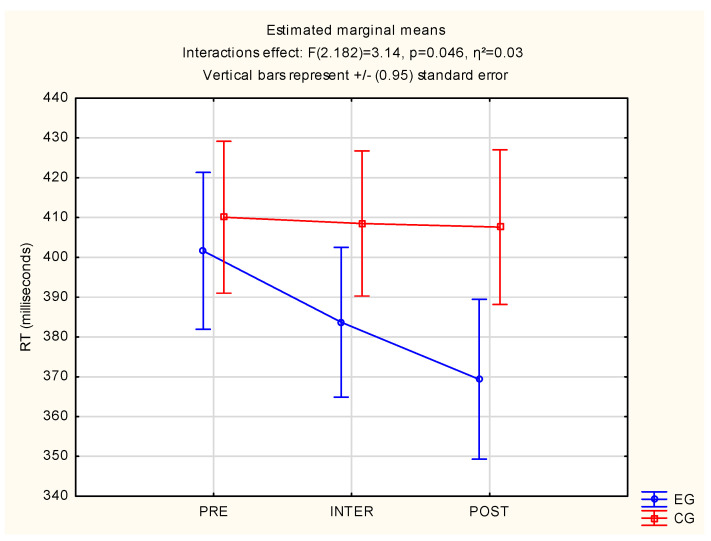
Statistical calculations between EG and CG of RT, for three measurements (PRE, INTER, POST). CG: control group; EG: experimental group; RT: reaction time.

Significant statistical interaction effects F(2.182) = 3.14, *p* = 0.046, η^2^ = 0.03 were observed for both groups in RT measurement. Differences between the EG and CG were found, where it was noted that the CG after a 30-min tabata-workout video had a longer RT (M = 409 ms) than the EG (M = 384 ms). In addition, the CG after the second 30-min tabata-workout video had a longer RT (M = 408 ms) than the EG (M = 369 ms). The above results are described in Figure 4.

**Figure 5:**
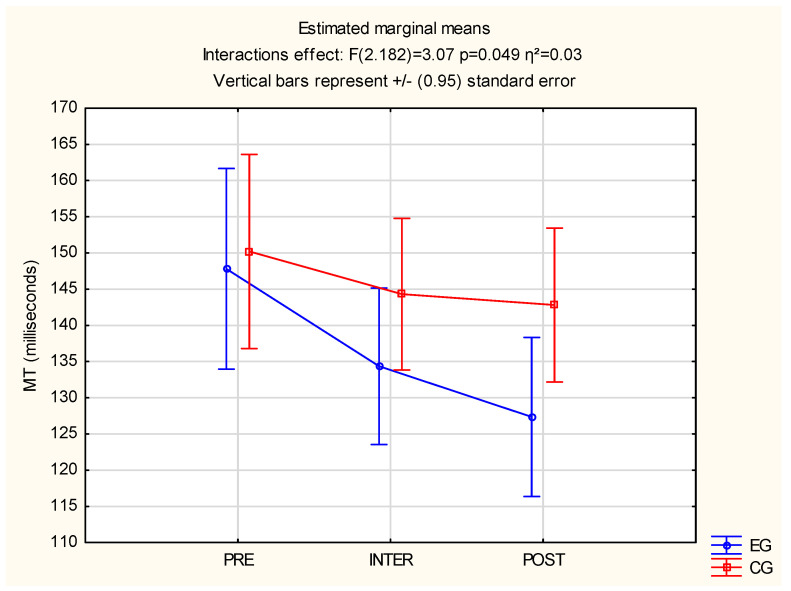
Statistical calculations between EG and CG of MT, for three measurements (PRE, INTER, POST). CG: control group; EG: experimental group; MT: motor time.

Significant statistical interaction effects F(2.182) = 3.07, *p* = 0.049 and η^2^ = 0.03 were observed for both groups in MT measurement. Differences between the EG and CG were found, where it was noted that the CG after a 30-min tabata-workout video had a longer MT (M = 144 ms) than the EG (M = 134 ms). Also, the CG after the second 30-min tabata-workout video had a longer MT (M = 143 ms) than the EG (M = 127 ms). The above results are described in Figure 5.

### 3.2. Postural Stability

**Figure 6:**
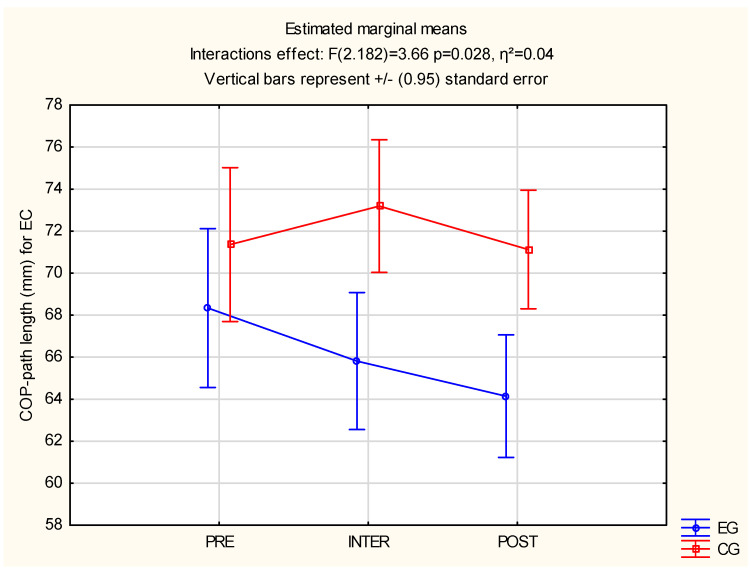
Statistical calculations between EG and CG of COP-path length for EC, for three measurements (PRE, INTER, POST). CG: control group; COP-path length: center of pressure path length; EC: EC-eyes close, EG: experimental group.

**Figure 7:**
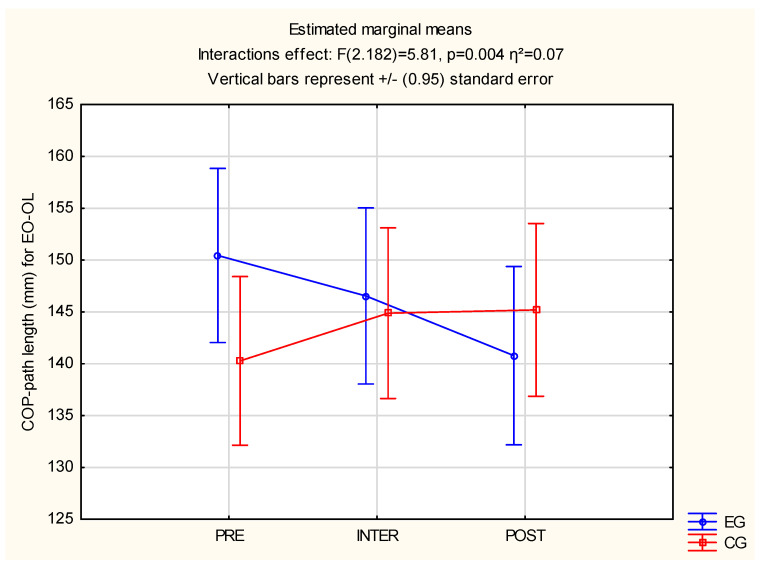
Statistical calculations between EG and CG of COP-path length for EO-OL, for three measurements (PRE, INTER, POST). CG: control group; COP-path length: center of pressure path length; EO-OL: eyes open and one leg, EG: experimental group.

Significant differences were observed between the groups in EC: F(2.182) = 3.66, *p* = 0.028 and η^2^ = 0.04 and EO-OL: F(2.182) = 5.81, *p* = 0.04 and η^2^ = 0.07. The above results are described in Table 4 and significant differences are available in Figure 6 and Figure 7.

**Figure 8:**
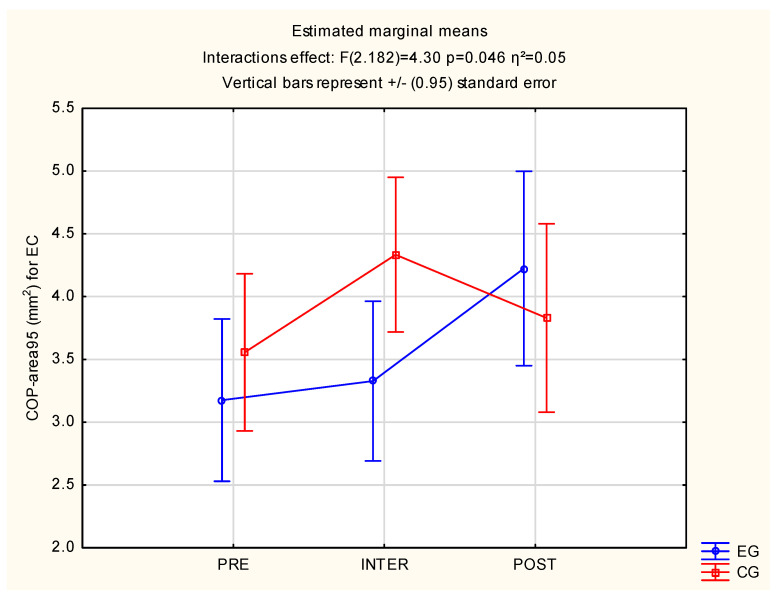
Statistical calculations between EG and CG of COP-area95 for EC, for three measurements (PRE, INTER, POST). CG: control group; COP-area95: center of pressure for area 95; EC: eyes close; EG: experimental group.

Significant differences were observed between the group in EC: F(2.182) = 4.30, *p* = 0.02 and η^2^ = 0.05. The above result is described in Table 5 and Figure 8.

### 3.3. Zephyr Measurement

An analysis of the results with ANOVA test shows the size of changes in mean values for the HR-Ave, HR-Max, BR-Ave, BR-Max and energy expenditure. Significant differences were observed between the groups in BR-Ave: F(2.182) = 2.65, *p* = 0.013 and η^2^ = 0.07; BR-Max: F(2.182) = 3.86, *p* = 0.02 and η^2^ = 0.04 and EE: F(2.182) = 3.84, *p* = 0.02 and η^2^ = 0.04.

## 4. Discussion

This study’s goals was to compare the effects of low-immersive vs. high-immersive exercise environment on postural stability, RT and MT in young adults. It was hypothesized that 1. high-immersive exercise environment would produce greater improvements in postural stability than low-immersive exercise environment. 2. High-immersive exercise environment would produce greater improvements in reaction and MT than low-immersive exercise environment. Our study confirmed both hypotheses.

Equipment used as a display for high-immersion can make a difference to postural stability. Soffel et al. [23] showed that postural stability measured with the VR head-mounted display (HMD) system in two conditions, eyes close and virtual fixation point’s distance during HMD wearing, are similar to each other. Image displays on a VR HMD screen for postural stability measurement can be compared to a condition with closed eyes. Thus, the VR environment is comparable to standing with eyes closed on a force platform, which probably can explain why eyes close measurements are better for the EG than CG, due to the similar conditions in our study. It is also worth noting that training using VR can eliminate the negative impact of visual stimuli on the level of postural stability [24]. Additionally, the sense of presence in the game, high immersion and the positive emotions associated with it helped to stabilize the standing on the force platform with EC. The mechanisms of enjoyment in gaming are a part of the technology’s ability to satisfy the basic psychological needs of the player, such as boosting feelings of competence, autonomy and relatedness [25]. VR exergames typically involve obstacle-based challenges and extrinsic reward systems to engage participants in interventions related to physical functioning [17]. Participants performed high-immersive in-game tasks, and they were able to transfer these positive emotions and competence to other tasks, such as a postural stability stand. Games are based on emotions and getting better and better results. It can be concluded that the positive emotions of a VR game can improve results in the tasks that follow. Immersion is the fundamental concept of the facilitation of emotion in a virtual environment. Some studies report an increase in emotional responses in more immersive compared to less immersive systems [15,16]. In addition, some studies emphasize that positive emotions promoted the learning effect and task performance [26].

Research on e-sport players showed that RT is affected by game playing time. If gamers play longer, the better RT they can have [27]. It is due to the speed of coding visual information into visual short-term memory, and the improvement does seem to depend on the time devoted to gaming [28]. In the case of our research, increased game time also improved better RT and MT. Another study show that exercise can improve the speed of reactions of young adults by energizing motor outputs but also higher RT disappears very quickly after the exercise cessation [29,30]. Additionally, RT can significantly be decreased for post-measurement, if a high-immersive system is applied [31,32,33]. In summary, the playing time for 60 min in total and the use of high immersion improves the results obtained in the RT and MT tests. The implementation of immersion resulted in reducing the time for RT and MT. However, training in a VR environment, which is a kind of high-immersion, gave the best final results for POST measurements.

Our study showed that exercise using higher immersion helps to increase postural stability and reducing the time for RT and MT compared to lower immersion system. This knowledge will encourage researchers to choose higher immersion, e.g., in the form of VR games instead of trainings displayed on a television screen as a remote interventions, in order to increase the results in this area of research.

## 5. Limitations and Future Directions

The limitation of this study was the acceptance of the necessity to be a physical education student as one of the inclusion criteria in this study. The aim was to study the effect on fully healthy, fit people. This allowed us to reduce the risk of the influence of other factors (e.g., poor health, chronic diseases, and low endurance). In addition, what we tried to highlight in the article, which mattered most, was the interest in the type of game and the gathering of the appropriate number of people for research who would meet the inclusion criteria, including the Ruffier squat test. To make it possible, we chose people interested in sports due to the type of studies selected. In the future, this study would be complemented by an analysis of the real processes that take place in the brain during the game.

The present study provides several directions for future studies in this research field. First of all, further studies are needed to examine better the high-immersive effects on postural stability, RT and MT, especially in different age groups, a population that hasn’t been investigated. In addition, high-immersive and low-immersive characteristics (e.g., genre, platform) in relation to postural stability, RT and MT skills should be further investigated in the future, in order to create specific and effective training programs.

## 6. Conclusions

The inclusion of higher immersion into an exercise environment produce greater improvements in MT and RT. Additionally, after a low-immersive exercise environment, participants have higher COP path length values with eyes close and standing with open eyes in one-leg tests, which testifies to less postural stability. Regarding COP trajectory, a smaller area surface means better performance for high-immersive participants, but only after 30 min of playing time. Increasing the workout to 60 min made the sway area worse than in CG. The high-immersive exercise environment can improve some aspects of postural stability and improve RT and MT.

## Figures and Tables

**Table 1:** Participants characteristics for age, body weight, body height, body mass index (BMI), female/male division (F/M), Ruffier index (RI).

Participants Characteristics	EG (95% CI)	CG (95% CI)
Age (years)	21.69 ± 2.76 (22.14)	22.25 ± 1.66 (21.77)
Body weight (kg)	71.03 ± 13.07 (74.96)	68.39 ± 12.96 (64.62)
Body height (cm)	173.98 ± 8.09 (176.41)	173.83 ± 8.63 (176.33)
BMI (kg/m^2^)	23.3 ± 3.23 (24.31)	22.5 ± 2.80 (23.28)
F/M (number)	22/23	26/22
RI (Ruffier index)^1^	6.5	6.8

**Table 2:** Exergames playlist for beginner and regular of Audio Trip game mode including title, duration and beats per minute.

Title (Chronological)	Duration (min:s)	BPM (Beats per Minute)
Dance monkey	03:28	98
Bangarang	03:32	110
Just Dance	03:38	118
Krishna	02:39	128
Red	07:31	136
Satisfaction	04:44	136
Mandala	04:39	143

**Table 3:** Tabata-video workout playlist including title, duration, and movement type.

Title (Chronological)	Duration (min:s)	Movement Type ^1^
Dance monkey ^2^	03:28	00:30 dumbbell fly’s ×3 ^2^ 00:30 boxing run ×4 ^2^
Bangarang ^3^	03:32	00:30 jabs left/right hand ×3 ^3^ 00:30 biceps drop down ×2 ^3^ 00:30 diagonally jabs ×2 ^3^
Just Dance ^4^	03:38	00:30 marking “X” ×2 ^4^ 00:30 knee up ×2 ^4^ 00:30 jumps left to right ×2 ^4^ 00:30 marking “square” ×1 ^4^
Krishna ^5^	02:39	00:30 CrossFit rope ×3 ^5^ 00:30 marking “u” in a squat ×2 ^5^
Red ^6^	07:31	00:30 jabs side to side ×3 ^6^ 00:30 dumbbell fly’s ×2 ^6^ 00:30 V-step ×3 ^6^ 00:30 marking “circle” ×2 ^6^ 00:30 squat wrists supination ×3 ^6^ 00:30 torso twists ×2 ^6^
Satisfaction ^7^	04:44	00:30 marking “wave” ×2 ^7^ 00:30 diagonally jabs side to side ×2 ^7^ 00:30 marking “wave” ×2 ^7^ 00:30 drums ×2 ^7^ 00:30 extra step ×2 ^7^
Mandala ^8^	04:39	00:30 squat dumbbell fly’s ×3 ^8^ 00:30 arms circling ×3 ^8^ 00:30 knee up to hand ×3 ^8^ 00:10 EXTRA: deep breath

^1^ After each sequence, a 00:10 step touch was performed as a form of active rest (imitation of the next track selection for Audio Trip). ^2,3,4,5,6,7,8^ Movement types for each music title.

**Table 4:** Changes in the path length of postural stability tests in three measurements (PRE, INTER, POST) in the CG and EG.

Path Length	CG	EG	Interaction
EO ^1^ (mm)	61.42	60.17	F(2.182) = 1.29, *p* = 0.28, η^2^ = 0.01
EO ^2^ (mm)	64.97	62.60	
EO ^3^ (mm)	66.19	62.81	
EC ^1^ (mm)	71.36	68.16	F(2, 182) = 3.66, *p* = 0.028 *, η^2^ = 0.04
EC ^2^ (mm)	72.49	66.55	
EC ^3^ (mm)	70.63	65.45	
Romberg ^1^	0.87	0.90	
Romberg ^2^	0.90	0.95	
Romberg ^3^	0.95	0.97	
EO-OL ^1^ (mm)	140.28	150.45	F(2, 182) = 5.81, *p* = 0.004 *, η^2^ = 0.07
EO-OL ^2^ (mm)	144.88	146.54	
EO-OL ^3^ (mm)	145.20	140.78	

^1,2,3^—number of measurements, *—significant differences. EO: eyes open, EC: eyes close, EO-OL: eyes open and one leg.

**Table 5:** Changes in the sway area of postural stability tests in three measurements (PRE, INTER, POST) in the CG and EG.

Area95	CG	EG	Interaction
EO ^1^ (mm^2^)	2.43	2.39	F(2, 182) = 0.60, *p* = 0.57, η^2^ = 0.01
EO ^2^ (mm^2^)	3.06	3.44	
EO ^3^ (mm^2^)	3.17	3.57	
EC ^1^ (mm^2^)	3.56	3.18	F(2, 182) = 4.30, *p* = 0.02 *, η^2^ = 0.05
EC ^2^ (mm^2^)	4.34	3.33	
EC ^3^ (mm^2^)	3.83	4.23	
EO-OL ^1^ (mm^2^)	8.25	9.24	F(2, 182) = 0.76, *p* = 0.47, η^2^ = 0.01
EO-OL ^2^ (mm^2^)	9.56	11.09	
EO-OL ^3^ (mm^2^)	10.67	11.09	

^1,2,3^—number of measurement, *—significant differences. EO: eyes open, EC: eyes close, EO-OL: eyes open and one leg.

## Data Availability

Not applicable.

## References

[1] Blaszczyk J.W., Hansen P.D., Lowe D.L. (1993). Postural Sway and Perception of the Upright Stance Stability Borders. Perception.

[2] Dunstan D.W., Howard B., Healy G.N., Owen N. (2012). Too Much Sitting—A Health Hazard. Diabetes Res. Clin. Pract..

[3] Hrysomallis C. (2011). Balance Ability and Athletic Performance. Sports Med..

[4] Iqbal K., Roy A. (2009). A Novel Theoretical Framework for the Dynamic Stability Analysis, Movement Control, and Trajectory Generation in a Multisegment Biomechanical Model. J. Biomech. Eng..

[5] Ołpińska-Lischka M., Kujawa K., Maciaszek J. (2021). Differences in the Effect of Sleep Deprivation on the Postural Stability among Men and Women. Int. J. Environ. Res. Public Health.

[6] Kenney E.L., Gortmaker S.L. (2017). United States Adolescents’ Television, Computer, Videogame, Smartphone, and Tablet Use: Associations with Sugary Drinks, Sleep, Physical Activity, and Obesity. J. Pediatr..

[7] Pallavicini F., Ferrari A., Mantovani F. (2018). Video Games for Well-Being: A Systematic Review on the Application of Computer Games for Cognitive and Emotional Training in the Adult Population. Front. Psychol..

[8] Okubo Y., Schoene D., Lord S.R. (2016). Step Training Improves Reaction Time, Gait and Balance and Reduces Falls in Older People: A Systematic Review and Meta-Analysis. Br. J. Sports Med..

[9] Liu R., Menhas R., Dai J., Saqib Z.A., Peng X. (2022). Fitness Apps, Live Streaming Workout Classes, and Virtual Reality Fitness for Physical Activity during the COVID-19 Lockdown: An Empirical Study. Front. Public Health.

[10] McDonough D.J., Helgeson M.A., Liu W., Gao Z. (2022). Effects of a Remote, YouTube-Delivered Exercise Intervention on Young Adults’ Physical Activity, Sedentary Behavior, and Sleep during the Covid-19 Pandemic: Randomized Controlled Trial. J. Sport Health Sci..

[11] Romeas T., More-chevalier B., Charbonneau M., Bieuzen F. (2022). Virtual-Reality Training of Elite Boxers Preparing for the Tokyo 2020 Olympics During the COVID-19 Pandemic: A Case Study. Case Stud. Sport Exerc. Psychol..

[12] Woyo E., Nyamandi C. (2021). Application of Virtual Reality Technologies in the Comrades’ Marathon as a Response to Covid-19 Pandemic. Dev. S. Afr..

[13] Martirosov S., Bureš M., Zítka T. (2021). Cyber Sickness in Low-Immersive, Semi-Immersive, and Fully Immersive Virtual Reality. Virtual Real..

[14] Nilsson N.C., Nordahl R., Serafin S. (2016). Immersion Revisited: A Review of Existing Definitions of Immersion and Their Relation to Different Theories of Presence. Hum. Technol..

[15] Kim A., Chang M., Choi Y., Jeon S., Lee K. The Effect of Immersion on Emotional Responses to Film Viewing in a Virtual Environment. Proceedings of the 2018 IEEE Conference on Virtual Reality and 3D User Interfaces (VR).

[16] Visch V.T., Tan E.S., Molenaar D. (2010). The Emotional and Cognitive Effect of Immersion in Film Viewing. Cogn. Emot..

[17] Tao G., Garrett B., Taverner T., Cordingley E., Sun C. (2021). Immersive Virtual Reality & Health Games: A Narrative Review of Game Design. J. NeuroEng. Rehabil..

[18] Eknoyan G. (2007). Adolphe Quetelet (1796 1874) the Average Man and Indices of Obesity. Nephrol. Dial. Transplant..

[19] Witmer B.G., Singer M.J. (1998). Measuring Presence in Virtual Environments: A Presence Questionnaire. Presence Teleoperat. Virtual Environ..

[20] Nagymáté G., Orlovits Z., Kiss R.M. (2018). Reliability Analysis of a Sensitive and Independent Stabilometry Parameter Set. PLoS ONE.

[21] Asseman F., Caron O., Crémieux J. (2005). Effects of the Removal of Vision on Body Sway during Different Postures in Elite Gymnasts. Int. J. Sports Med..

[22] Tjernström F., Björklund M., Malmström E.-M. (2015). Romberg Ratio in Quiet Stance Posturography—Test to Retest Reliability. Gait Posture.

[23] Soffel F., Zank M., Kunz A. Postural Stability Analysis in Virtual Reality Using the HTC Vive. Proceedings of the 22nd ACM Conference on Virtual Reality Software and Technology.

[24] Cyma-Wejchenig M., Tarnas J., Marciniak K., Stemplewski R. (2020). The Influence of Proprioceptive Training with the Use of Virtual Reality on Postural Stability of Workers Working at Height. Sensors.

[25] Tamborini R., Grizzard M., David Bowman N., Reinecke L., Lewis R.J., Eden A. (2011). Media Enjoyment as Need Satisfaction: The Contribution of Hedonic and Nonhedonic Needs. J. Commun..

[26] Tan J., Mao J., Jiang Y., Gao M. (2021). The Influence of Academic Emotions on Learning Effects: A Systematic Review. Int. J. Environ. Res. Public Health.

[27] Ersin A., Tezeren H.C., Pekyavas N.O., Asal B., Atabey A., Diri A., Gonen İ. The Relationship between Reaction Time and Gaming Time in E-SPORTS Players. https://hrcak.srce.hr/ojs/index.php/kinesiology/article/view/19294.

[28] Wilms I.L., Petersen A., Vangkilde S. (2013). Intensive Video Gaming Improves Encoding Speed to Visual Short-Term Memory in Young Male Adults. Acta Psychol..

[29] Davranche K., Audiffren M., Denjean A. (2006). A Distributional Analysis of the Effect of Physical Exercise on a Choice Reaction Time Task. J. Sports Sci..

[30] Audiffren M., Tomporowski P.D., Zagrodnik J. (2008). Acute Aerobic Exercise and Information Processing: Energizing Motor Processes during a Choice Reaction Time Task. Acta Psychol..

[31] Pallavicini F., Pepe A. (2020). Virtual Reality Games and the Role of Body Involvement in Enhancing Positive Emotions and Decreasing Anxiety: Within-Subjects Pilot Study. JMIR Serious Games.

[32] Glueck A.C., Han D.Y. (2019). Improvement Potentials in Balance and Visuo-Motor Reaction Time after Mixed Reality Action Game Play: A Pilot Study. Virtual Real..

[33] Gumaa M., Khaireldin A., Rehan Youssef A. (2021). Validity and Reliability of Interactive Virtual Reality in Assessing the Musculoskeletal System: A Systematic Review. Curr. Rev. Musculoskelet. Med..

